# The Amplatzer device and pedicle muscle flap transposition for the treatment of bronchopleural fistula with chronic empyema after lobectomy: two case reports

**DOI:** 10.1186/s12957-021-02270-x

**Published:** 2021-05-26

**Authors:** Yongyong Wu, Zhongliang He, Weihua Xu, Guoxing Chen, Zhijun Liu, Ziying Lu

**Affiliations:** 1 Department of Cardiothoracic Surgery, Tongde Hospital of Zhejiang Province, Hangzhou, 310012 Zhejiang China; 2 Department of Respiratory Medicine, Tongde Hospital of Zhejiang Province, Hangzhou, 310012 Zhejiang China; 3 Department of General Surgery, Tongde Hospital of Zhejiang Province, Hangzhou, 310012 Zhejiang China

**Keywords:** Bronchopleural fistula, Amplatzer device, Chronic empyema, Muscle flap transposition

## Abstract

**Background:**

Bronchopleural fistula (BPF) refers to an abnormal channel between the pleural space and the bronchial tree. It is a potentially fatal postoperative complication after pulmonary resection and a complex challenge for thoracic surgeons because many patients with BPF ultimately develop refractory empyema, which is difficult to manage and has a major impact on quality of life and survival. Therefore, an operative intervention combined with conservative and endoscopic therapies may be required to control infection completely, to occlude BPF, and to obliterate the empyema cavity during treatment periods.

**Case presentation:**

Two patients who suffered from BPF complicated with chronic empyema after lobectomy were treated in other hospitals for a long time and did not recover. In our department, we performed staged surgery and creatively combined an Amplatzer Septal Occluder (ASO) device (AGA Medical Corp, Golden Valley, MN, USA) with pedicled muscle flap transposition. First, open-window thoracostomy (OWT), or effective drainage, was performed according to the degree of contamination in the empyema cavity after the local infection was controlled. Second, Amplatzer device implantation and pedicled muscle flap transposition was performed at the same time, which achieved the purpose of obliterating the infection, closing the fistula, and tamponading the residual cavity. The patients recovered without complications and were discharged with short hospitalization stays.

**Conclusions:**

We believe that the union of the Amplatzer device and pedicle muscle flap transposition seems to be a safe and effective treatment for BPF with chronic empyema and can shorten the length of the related hospital stay.

## Introduction

With the continuous improvement of surgical techniques and perioperative management, the incidence of bronchopleural fistula (BPF) after lung resection has gradually decreased to approximately 0.5–5.0% in recent years; however, once BPF occurs, the mortality rate can be as high as 16–71% [1]. The treatment of BPF after lung resection needs to be individualized. For early BPF, conservative treatment or scheduled therapeutic reoperation can be considered to repair the fistula. For the middle and late stages of BPF combined with chronic empyema, integrated treatments are necessary, including conservative, surgical, and bronchoscopy treatment. The comprehensive application of various stents or blocking materials or stage one open-window thoracostomy (OWT) and thoracoplasty are adopted, and the second stage uses intrathoracic muscular transposition to obliterate the residual cavity [2, 3]. Endoscopic Amplatzer device implantation is an emerging technology that has been reported to be used in the treatment of large BPF in recent years, and its effectiveness is gradually being confirmed [4]. However, up to the present time, there has not been a simultaneous use of the Amplatzer device and pedicle muscle flap transposition for the treatment of BPF with chronic empyema reported in the English-language literature. In this article, we present the potential advantages of this hybrid technique of interdisciplinary teamwork and provide a new choice for similar cases.

## Case report

### Case 1

An 82-year-old male with a history of hypertension and diabetes underwent thoracoscopic right lower lobectomy and mediastinal lymph node dissection for pT2N0M0 squamous cell carcinoma in February 2014 and did not receive adjuvant radiotherapy and chemotherapy post-operation. Three years later, he was readmitted to the hospital with persistent cough and fever. Chest computerized tomography (CT) and bronchoscopy showed bronchial stump fistula and empyema, and closed-tube thoracostomy was performed for drainage, but the therapeutic effect was unsatisfactory. The bronchial stent graft was implanted under the bronchoscope in May 2017, and the purulent cavity was continuously irrigated and drained; however, there was still a foul smell and purulent secretion flowing out from the abscess cavity.

**Fig. 1:**
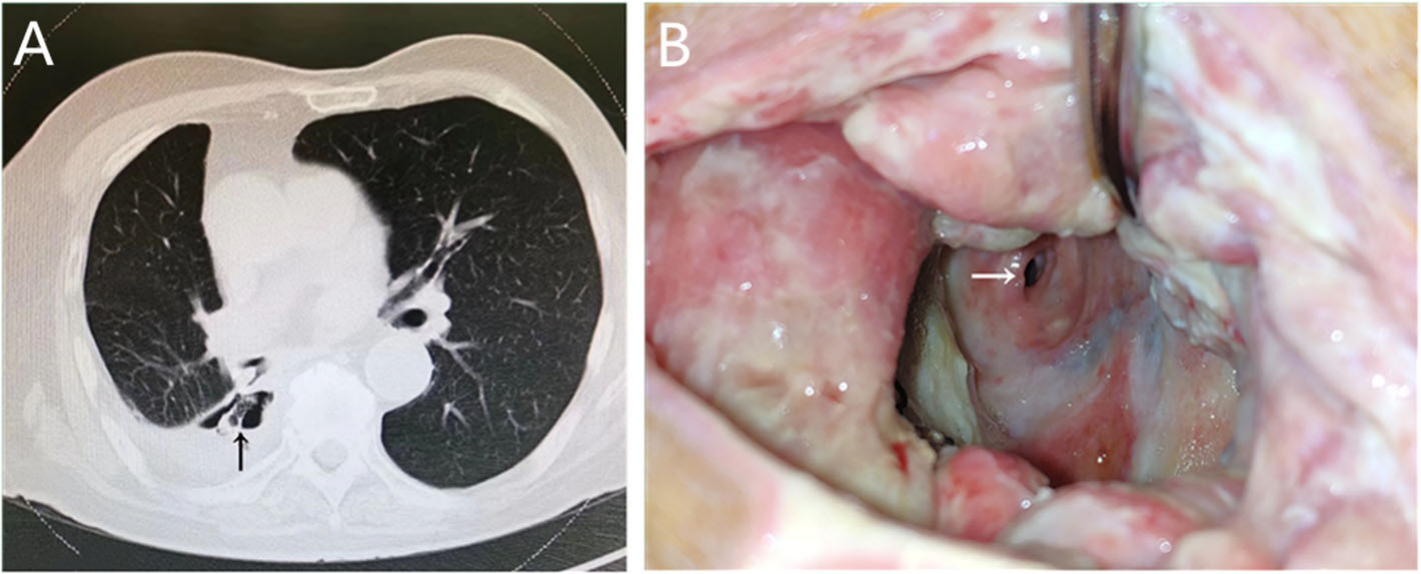
**A** Chest CT shows that the bronchial stent was displaced and dropped into the abscess cavity (black arrow). **B** After changing the dressings for 4 weeks, the cavity wall was clean, and the granulation tissue was fresh. A BPF with a diameter of approximately 8 mm can be seen (white arrow)

In July 2019, the patient was transferred to our department for hospitalization. Positron emission tomography (PET)-CT examination showed no evidence of local recurrence or distant metastasis of the tumor, and the bronchial stent was displaced and dropped into the abscess cavity (Fig. 1A). An exploratory thoracotomy was performed through a muscle-sparing incision; one segment of the rib approximately 8 cm long was removed during the operation, the necrotic tissue was obliterated, the dimension of the residual cavity (approximately 80 cm^3^) and the number and size of fistulas were measured, and the stent was removed. Bacterial culture revealed *Pseudomonas aeruginosa*, and then a modified OWT was performed because of the severe infection of the empyema cavity. After dressings with iodoform gauze were performed daily for 4 weeks, the infection was controlled, and fresh granulation tissue was present in the cavity. Meanwhile, a bronchial stump fistula approximately 8 mm in diameter was clearly visible (Fig. 1B).

**Fig. 2:**
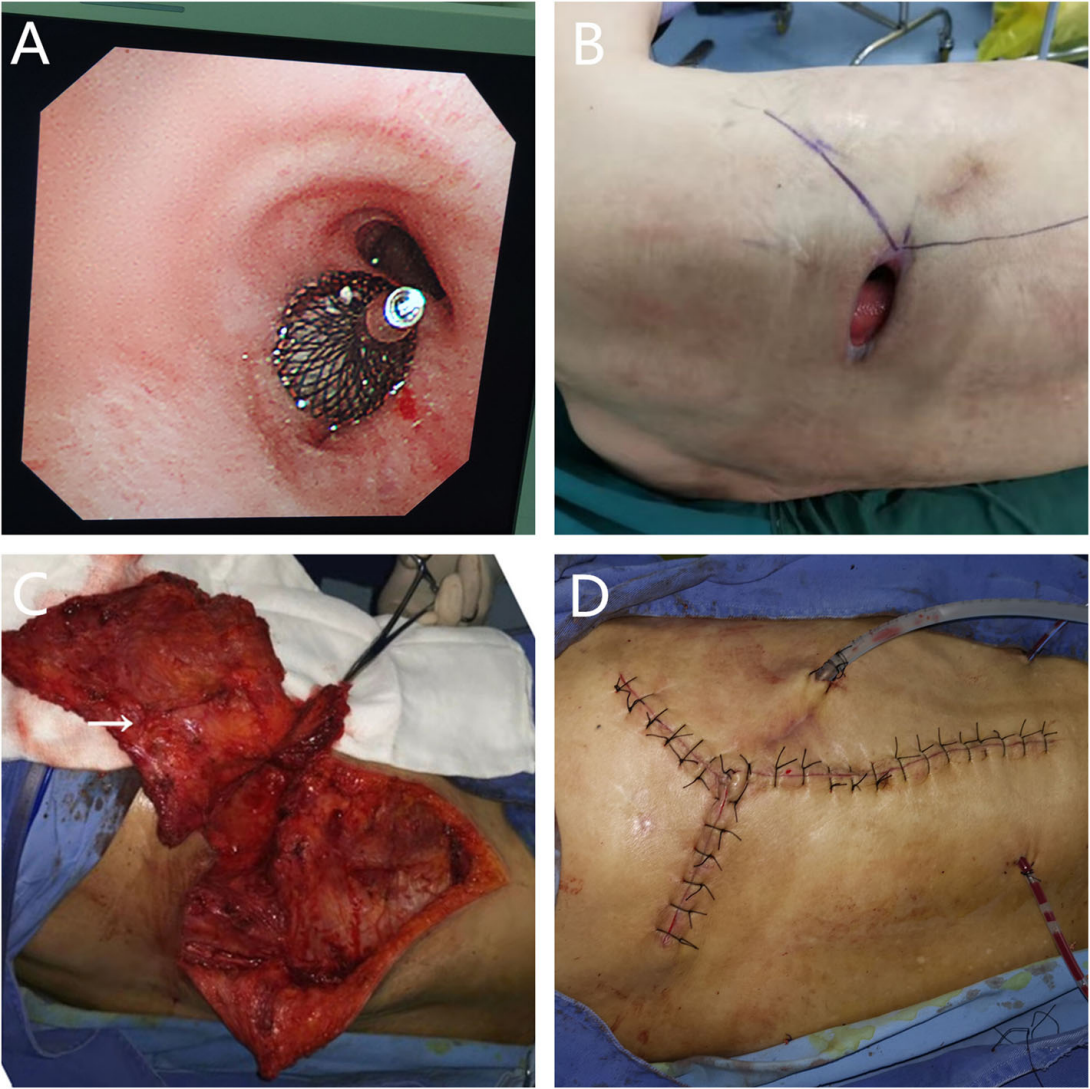
**A** The Amplatzer device was implanted to completely occlude the fistula in a good position. **B** Preoperative incision. **C** The completely separated latissimus dorsi muscle flap pedicled with the dorsal thoracic artery (arrow). **D** The incision after an operation

**Fig. 3:**
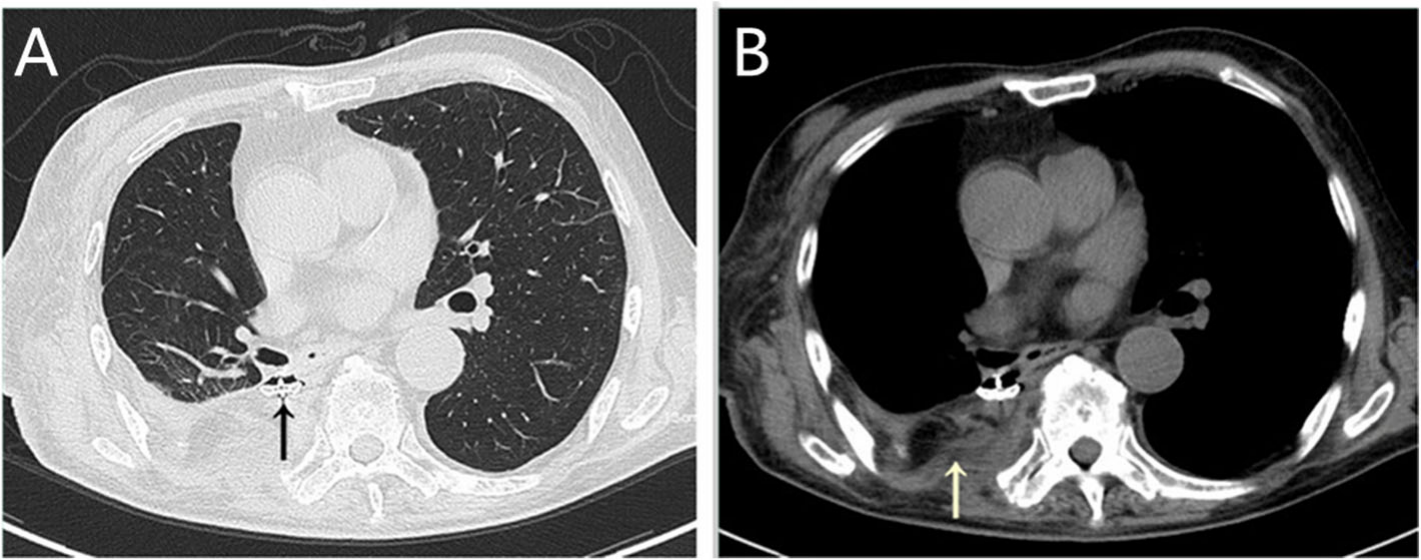
**A** The occluder had slipped into the thorax. **B** The transferred muscle flap filled the residual cavity

The simultaneous operation was performed in August 2019. The patient was placed in the 90° lateral position under general anesthesia with double-lumen endotracheal intubation. First, the respiratory physician completed the Amplatzer device (Amplatzer Septal Occluder (ASO); waist diameter, 8 mm; AGA Medical Corp, Golden Valley, MN, USA) implantation through the bronchoscope, the fistula was blocked absolutely, and the position of the occluder was fine (Fig. 2A). Then, cardiothoracic surgeons performed intrathoracic muscular transposition, and an axillary incision to the anterior superior iliac spine was made to separate the latissimus dorsi muscle flap adequately (Fig. 2B). Regarding the necrotic cavity decortication, the cavity was irrigated with a large amount of hydrogen peroxide, normal saline, and povidone-iodine solution. The thoracic dorsal artery and vein were used as the pedicle and were anatomically separated to the proximal end of the blood vessel, thus forming a latissimus dorsi muscle flap with the neurovascular bundle (Fig. 2C). The residual cavity was filled, and the muscle flap was properly fixed to the chest wall. A chest and several subcutaneous drainage tubes were placed near the fistula, and the incision was closed (Fig. 2D). The patient recovered well and was discharged 2 weeks postoperatively. At the 15-month follow-up, he remained well and showed no signs of BPF or empyema recurrence. On the CT scan, although the occluder had slipped into the thorax (Fig. 3A), the transferred muscle flap filled the residual cavity (Fig. 3B); however, the patient was asymptomatic.

### Case 2

**Fig. 4:**
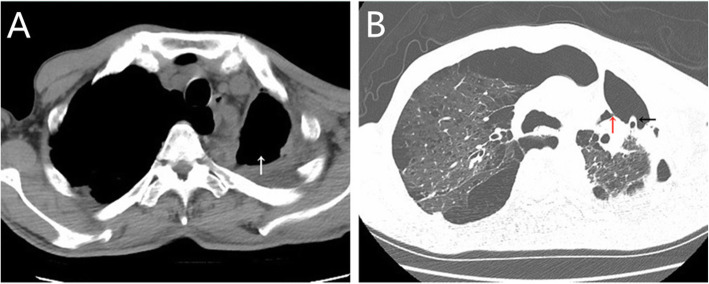
**A** The residual cavity at the apex of the left chest. **B** A bronchial stump fistula (red arrow) and chest tube (black arrow)

**Fig. 5:**
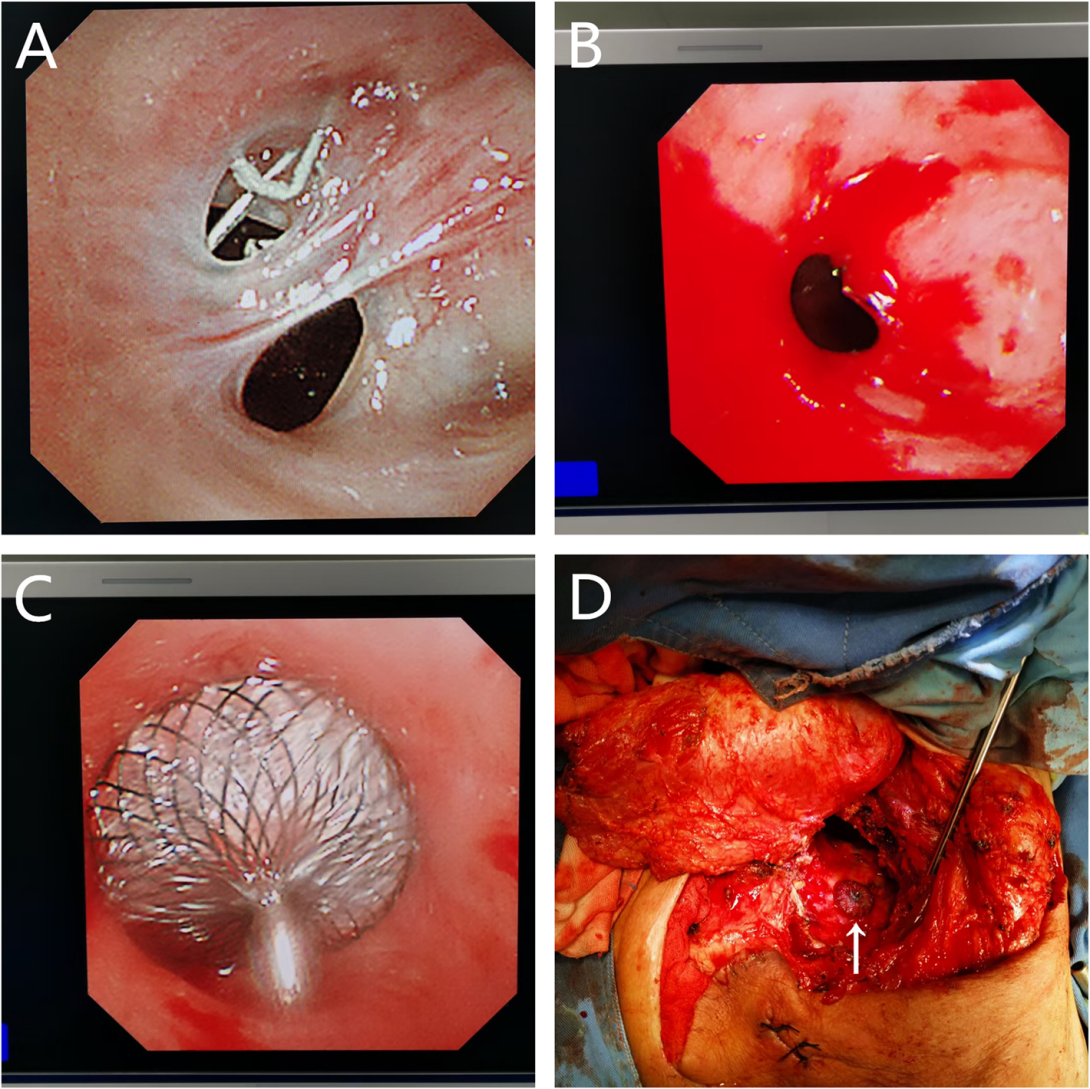
**A** bronchial stump fistula with separation and residual suture nails can be seen under electronic bronchoscopy. **B** After cutting and ablation, the fistula with a diameter of 7 mm was completely exposed. **C** The Amplatzer occluder was implanted to seal the fistula completely under bronchoscopy. **D** The satisfactory plugging effect of the Amplatzer device is shown under direct vision (arrow)

A 58-year-old male with a history of diabetes underwent video-assisted thoracoscopic left upper lobectomy for pT3N0M0 stage IIb squamous cell carcinoma in January 2013, followed by 4 courses of adjuvant chemotherapy. Seven years later, the patient was readmitted for persistent cough and purulent secretion exudation from the chest wall incision. Chest CT (Fig. 4A) and electronic bronchoscopy confirmed bronchial stump fistula (Fig. 5A) with empyema. Then, a chest drainage tube (Fig. 4B) was placed, and approximately 50 ml of off-white liquid was drawn out daily. After 3 months of unimproved treatment, he was transferred to our hospital for further therapy.

**Fig. 6:**
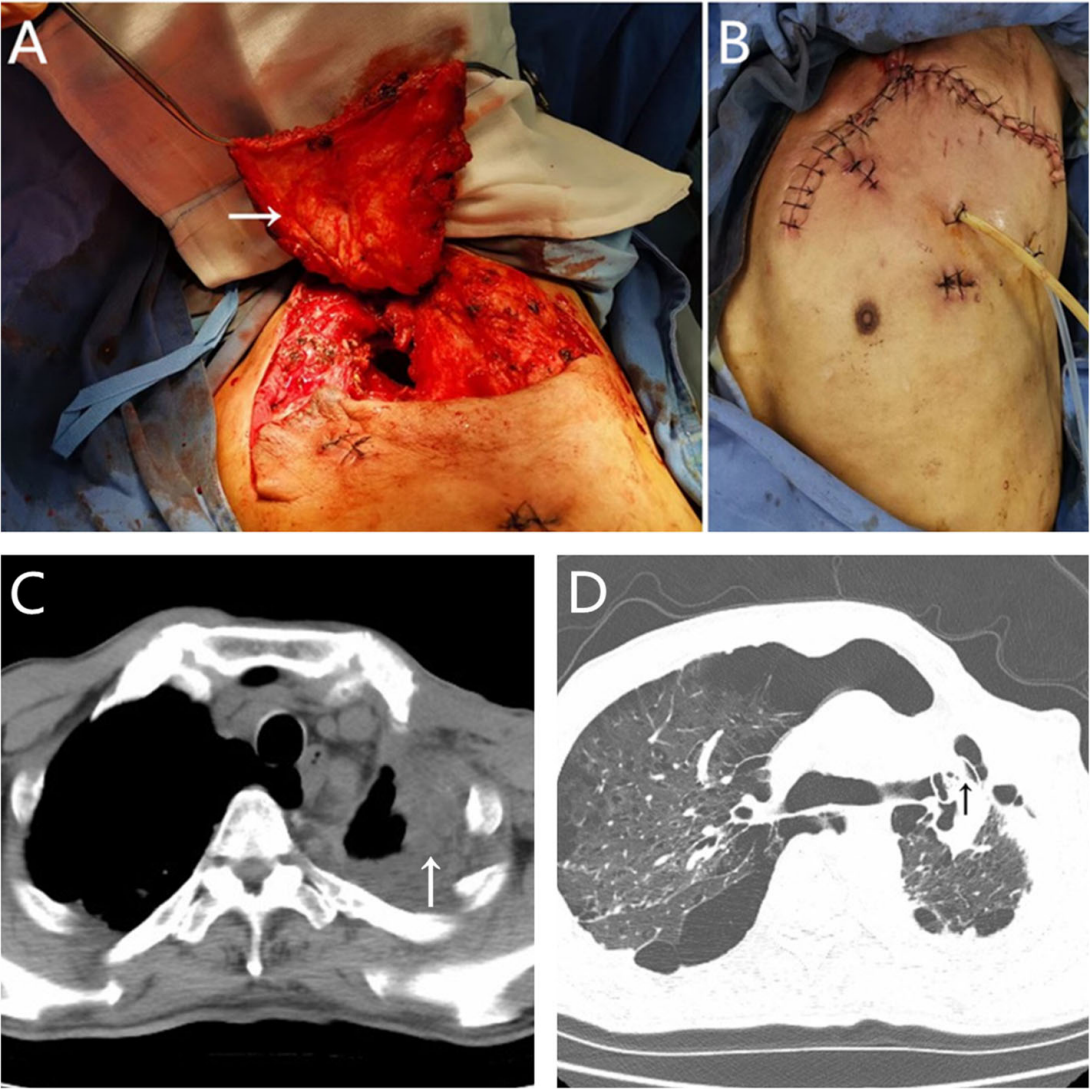
**A** The completely separated pectoralis major muscle flap pedicled with the descending branch of the thoracoacromial artery (arrow). **B** The incision after the operation. **C** The residual cavity at the apex of the chest disappeared. **D** The occluder was in a good position

An exploratory thoracotomy was performed through a minimal incision and found that the dimension of the abscess cavity was approximately 60 cm^3^. Bacterial culture revealed the presence of *Klebsiella pneumoniae*. Effective drainage was performed according to mild contamination in the empyema cavity. When the infection of the cavity was controlled, a hybrid operation was performed in May 2020, and the procedure was the same as that used in case 1. The laser cut the tissue band between the fistula and ablated the suture nail under the bronchoscope, and the diameter of the fistula was approximately 7 mm after full exposure (Fig. 5B). The Amplatzer device (ASO; waist diameter, 10 mm; AGA Medical Corp, Golden Valley, MN, USA) was implanted successfully (Fig. 5C), and the occlusion effect was satisfactory under direct view (Fig. 5D). According to the position of the residual cavity, the pectoralis major muscle flap was adequately separated from the descending branch of the thoracic acromion artery (Fig. 6A), and the incision was sutured after sufficient filling (Fig. 6B). The patient recovered uneventfully and was discharged 2 weeks post-operation. At the 6-month follow-up, he was asymptomatic and showed no evidence of recurrence. On the CT scan, we observed that the residual cavity at the apex of the chest was filled with a muscle flap (Fig. 6C) and that the occluder was in a good position (Fig. 6D).

## Discussion

BPF is one of the most serious complications after lung resection and a difficult problem faced by thoracic surgeons today because of tricky treatment and poor results. A multivariate analysis of risk factors for BPF reported by Asamura et al. identified lobectomy, mediastinal lymph node dissection, high-dose preoperative radiation therapy, and recurrent carcinoma in the bronchial stump as technical factors predisposing patients to BPF; nonoperative factors included diabetes mellitus, cirrhosis, hypoalbuminemia, and steroid use [5]. There are two important aims of treatment; one is to control the infection and to close the fistula, and the other is to obliterate the empyema space. Options include conservative, endoscopic, and surgical treatments [6].

Conservative therapy is simple, safe, and non-invasive, including supportive treatments and effective drainages. Adequate pleural drainage remains the cornerstone of empyema management, and different drainage methods have been described. Closed chest tube drainage is advocated as the first step in the treatment of chronic empyema, but the high failure rate demonstrates that it cannot control infection of the residual cavity effectively and increases the risk of aspiration pneumonia and death [7, 8]. OWT has proven to be a very useful and simple technique that provides adequate control of empyema after total and partial lung resection. This approach, however, can lead to poor quality of life because of long-term dressing changes, compressive bandages, poor quality of life, and chronic thoracic pain [9]. Endoscopic therapy should be performed to promote fistula closure after thoracic drainage because fistula closure is the key to a successful surgical outcome. This procedure should include the bronchial mechanical abrasion, submucosal injection, endoscopic placement of biological glue, coils, and silver nitrate, and the placement of covered stents, endobronchial valves, and an Amplatzer device. The degree of endoscopic success is variable and depends on the patient’s underlying disease and the proximity and size of the fistulas, and the success rate is 22.5 to 96.9% [10]. Meanwhile, endoscopic treatment can be used as a bridge to control infection and to create conditions for surgical treatment or as a hybrid technique for a BPF greater than 6 mm in diameter [11].

An Amplatzer device is commonly used for transcatheter closure of atrial septal defects. It has a double-disc design with a waist connection in the middle and is woven with a superelastic nickel-titanium alloy wire, and it has good biocompatibility and can promote intrabronchial granulation tissue. Growth improves the sealing effect and reduces the risk of displacement. When sealing the fistula, the waist is placed inside the fistula, and two discs are placed at the proximal and distal ends of the fistula [12]. Fruehter et al. reported 31 cases of BPF treated with the Amplatzer device in which the clinical symptoms of the postoperative patients were immediately improved and long-term follow-up was performed. No serious adverse reactions occurred, and the success rate of fistula closure was high [13]. However, because the original design does not fully conform to the physiological environment of the respiratory tract, other combined treatment methods are necessary as supplements according to different conditions and fistulas.

For chronic empyema, three pathologic conditions normally occur regardless of the cause: persistent residual cavity, cavity infection, and BPF. Intrathoracic transposition of the extrathoracic skeletal muscle or the pedicled omental flap can effectively eliminate the empyema and residual cavity [14, 15], which mainly include the latissimus dorsi, serratus anterior, pectoralis major, rectus abdominis, and pedicled omental flap. The flap tissue is large, strong in anti-infection ability, and effective in packing. When autologous muscle flap transposition is used to treat chronic refractory empyema, it is best to ensure that the bacterial culture in the residual cavity is negative, healthy new granulation tissue is covered with the residual cavity, exudates are reduced, and the general condition of the patient is improved [16]. For these two patients, thoracotomy was performed to explore the infectious cavity and to measure both the volume of the abscess cavity and the number and size of the fistulas. Then, the OWT or productive tube drainage procedure was chosen according to the degree of infection in the empyema cavity. Through a period of conservative and systemic supportive treatment, when the granulation tissue of the abscess cavity wall was fresh, the temperature and leukocytes were normal, tube drainage fluid remained clear, and bacterial culture was negative. Both patients underwent simultaneous surgery with Amplatzer device implantation combined with pedicle muscle flap transposition.

Based on our previous clinical experience [6, 9] and elaborate communication with respiratory physicians, we believe that it is feasible to perform Amplatzer closure and pedicle muscle flap transposition during the same surgical operation period. The main advantages of simultaneously combined surgery are as follows: (1) Shortening the course of the disease and hospitalization stays greatly reduces the suffering of long-term drainage, recurrent infections, and the frequent changing of dressings. (2) The existence of BPF is one of the critical failure factors of muscle flap transposition. An Amplatzer device can effectively close the larger fistula, fully isolate air and liquid, and provide basics for intrathoracic muscular transposition. The filled muscle tissue flap has good anti-infection ability and reduces the incidence of occluder-related complications; the two complement each other. (3) Through a simultaneous operation, the infection can be obliterated, the fistula can be closed, the residual cavity can be tamponaded, primary healing can be achieved, and the recurrence of pleural fistula and empyema can be greatly decreased.

## Conclusion

The treatment of BPF accompanied by chronic empyema is difficult, time-consuming, and costly. Finding a therapeutic schedule with a higher cure rate and less injury has always been the intention of cardiothoracic surgeons. In the English-language literature, previously, there has been no report of using an Amplatzer device and pedicled muscle flap transposition to manage BPF with chronic empyema. Based on the two abovementioned successful cases, we believe that the union of the two technologies seems to be safe and effective and can shorten the length of hospital stays. We also hope to prove that this hybrid technique can reduce the morbidity and mortality associated with reinfection and respiratory failure by further studies.

## Data Availability

Not applicable.

## Abbreviations

BPF: Bronchopleural fistula
OWT: Open-window thoracostomy
CT: Computerized tomography
PET: Positron emission tomography
ASO: Amplatzer Septal Occluder
